# A new concept of the fiber composition of cervical spinal dura mater: an investigation utilizing the P45 sheet plastination technique

**DOI:** 10.1007/s00276-022-02962-3

**Published:** 2022-06-17

**Authors:** Jing Zhuang, Jin Gong, Gary D. Hack, Yan-Yan Chi, Yang Song, Sheng-Bo Yu, Hong-Jin Sui

**Affiliations:** 1 Department of Anatomy, College of Basic Medicine, Dalian Medical University, 9 West Section, Lushun South Road, Dalian, 116044 People’s Republic of China; 2 Department of Advanced Oral Sciences and Therapeutics, University of Maryland School of Dentistry, Baltimore, MD 21201 USA; 3 Expert Workstation, Dalian Hoffen Preservation Technique Institution, Dalian, 116052 China

**Keywords:** Spinal dura mater, Craniovertebral junction, Microanatomy, P45 plastination

## Abstract

**Purpose:**

Few reports have been published regarding the microanatomy of the dura mater located at the craniovertebral junction (CVJ). In clinic, the precise microanatomy of the CVJ dura mater would be taken into account, for reducing surgical complications and ineffective surgical outcomes. The main objective of the present investigation was to further elucidate the fiber composition and sources of the cervical spinal dura mater.

**Methods:**

The formalin-fixed adult head and neck specimens (*n* = 21) were obtained and P45 plastinated section method was utilized for the present study. The fibers of the upper cervical spinal dura mater (SDM) were examined in the P45 sagittal sections in the CVJ area. All photographic documentation was performed via a Canon EOS 7D Mark camera.

**Results:**

The posterior wall of the SDM sac at CVJ was found to be composed of stratified fibers, which are derived from three sources: the cerebral dura mater, the occipital periosteum, and the myodural bridge (MDB). The proper layer of the cerebral dura mater passes over the brim of the foramen magnum and enters the vertebral canal to form the inner layer of the SDM, and the fibers originating from the periosteum of the brim of the foramen magnum form the middle layer. The fibers of the MDB are inserted into the SDM and form its outer layer. It was found that the total number of fibers from each origin varied in humans.

**Conclusion:**

At the CVJ, the posterior wall of the SDM is a multi-layered structure composed of three different originated fibers. The cerebral dura mater, the periosteum located at the brim of the foramen magnum, and MDB contribute to the formation of the SDM. The present study would be beneficial to the choice of surgical approach at the CVJ and the protection of the SDB.

## Introduction

It has previously been postulated that the spinal dura mater (SDM) is a direct extension of the cerebral dura mater and is closely associated with the occipital periosteum around the foramen magnum [7, 13, 20]. Our previous research observed that the rectus capitis posterior minor muscle (RCPmi) is connected to the upper cervical dura mater via a dense connective tissue bridge, the so-called myodural bridge (MDB). Moreover, we previously observed that the MDB contributes to the composition of the SDM [16, 21, 30, 32], which has not been evidenced in other studies. Recent research describing the composition of dura mater at the craniovertebral junction (CVJ) confirmed that the cranial dura mater’s outer layer is continuous with the dura mater’s outer layer in the spinal region. Moreover, the dura mater located at the CVJ displayed a dynamic morphological change within an extremely limited short segment [13]. We speculate that more sources of fibers would be involved in the dura mater at the CVJ. However, the anatomic basis for this morphological change and the fibrous origin of the cervical dura mater are not well described in the current literature. Although there have been many studies on the mechanical properties, blood vessels, and nerves associated with the SDM [6, 17, 29], few studies to date have described the microanatomy of the dura mater at the CVJ. In the present study, median and paramedian sagittal slices of the P45 plastinated specimens were observed to clarify the fiber composition of the cervical dura mater. P45 sheet plastination is a new type of plasticization technology. Specimens produced by this technology can reduce the problem of material shrinkage and keep the specimen in its natural state, which helps us to observe the morphology and running form of fibers more accurately. The observation of the source of dural fibers in this study may have a guiding role in the future dura dissection in cerebellar tonsillar hernia surgery and Chiari malformation type I duraplasty, and may avoid the occurrence of surgical complications such as cerebrospinal fluid leakage.

## Materials and methods

The formalin-fixed adult head and neck specimens (*n* = 21) were obtained from the Body and Organs Donation Center of Dalian Medical University for the present study. The P45 plastinated slices were prepared at the Dalian Hoffen Preservation Technique Institution. Median and paramedian sagittal slices of the P45 plastinated specimens (3 sections in each specimen, total 36 slices) were examined and recorded by photography. No abnormal anatomic variation, trauma, inflammation or mass lesions were observed in any of the specimens. Therefore, all specimens were included for observation.

The slice thickness was 3 mm. The condensation rate of the P45 plastinated sections was lowered and the original position of the tissues was maintained. The procedures involved in the P45 sheet plastination process included slicing, bleaching, dehydration, forced impregnation, and curing [10, 25, 33].

### Slicing

Embalmed head face specimens were frozen at -70℃ for 2 weeks. Then, 3-mm-thick sagittal slices were prepared with a high-speed band saw.

### Bleaching

All slices were rinsed with running water for 6–8 h and immersed in 5% hydrogen peroxide overnight for bleaching.

### Dehydration

After bleaching, the slices were dehydrated with 100% acetone by the freeze substitution method.

### Casting and forced impregnation

After dehydration, the casting mold was prepared. The slices were lifted from the acetone bath and placed between two glass plates. The molds were then filled with polyester (Hoffen polyester P45, Dalian Hoffen Bio-Technique Co. Ltd., Dalian, P. R. China,). The filled mold was then placed upright into a vacuum chamber at room temperature for impregnation. The large bubbles on the surface of the slices were removed manually with a 1-mm stainless steel wire. The absolute pressure was slowly decreased to 20, 10, 5, and 0 mmHg, according to the bubble release. The pressure was maintained at 0 mmHg until bubbling ceased. The impregnation step lasted for more than 8 h.

### Curing

After vacuum release, the top of the mold was clamped with large fold back clamps, and the sheet was ready for curing. The sheets were placed in an upright position and cured in a heated water bath at 40℃ for 3 days. After curing, the slices were taken out from a flat chamber and covered with adhesive plastic wrap for protection. A small band saw was used to cut and trim the plastic along the outside edges of the slices at a distance of approximately 1 mm from the tissue. To cut out the sharp edges of the slices, a wool sander was used. The sheets were then ready for use.

## Results

The cerebral dura mater, SDM, atlas (C1), axis (C2), RCPmi muscle, and the MDB were all clearly observed in the sagittal P45 plastinated slices of the head and neck.

### Three fibrous sources of the SDM at the CVJ

**Fig. 1:**
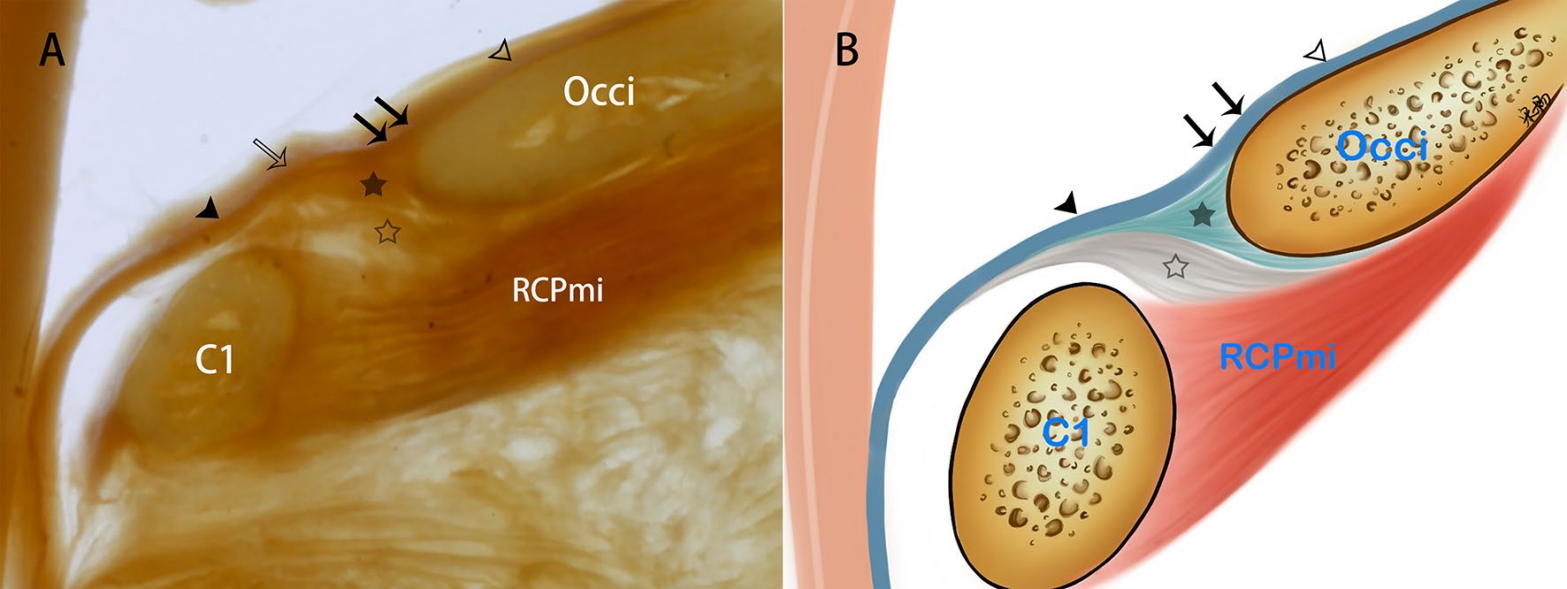
Images evidencing the three sources of the distinct fibers composing the spinal dura mater and an illustrative diagram. Occi, occipital bone; RCPmi, the rectus capitis posterior minor muscle; C1, atlas; hollow arrowhead, cerebral dura mater; solid arrowhead, spinal dura mater; double arrow, spinal dura mater; solid star, the fibers from the occipital periosteum; hollow star, the MDB; hollow arrow, the thickened spinal dura mater

In all of the P45 plastinated slices, it was observed that in the posterior cranial fossa, the cerebral dura mater extended into the vertebral canal, while passing over the brim of foramen magnum, to become the inner layer of the spinal dura mater (Fig. 1). Several fibers were observed to originate from the outer periosteum located on the inferior surface of the brim of the foramen magnum. These fibers coalesced and ultimately fused with the inner layer of the spinal dura mater to form the middle layer of the spinal dura mater (Fig. 1). Also, the RCPmi muscle was observed to send out dense connective fibers, namely the myodural bridge (MDB) which has been described in previous studies [8], which merged into the spinal dura mater while passing through the posterior atlanto-occipital interspace to form the outer layer of the SDM (Fig. 1). It was also found that in the sagittal plastinated sections of the CVJ, the spinal dura mater at the level of the posterior atlanto-occipital interspace was quite thick in comparison with the dura mater in the posterior cranial fossa (Fig. 1A).

### Structural characteristics of the three components of the SDM

**Fig. 2:**
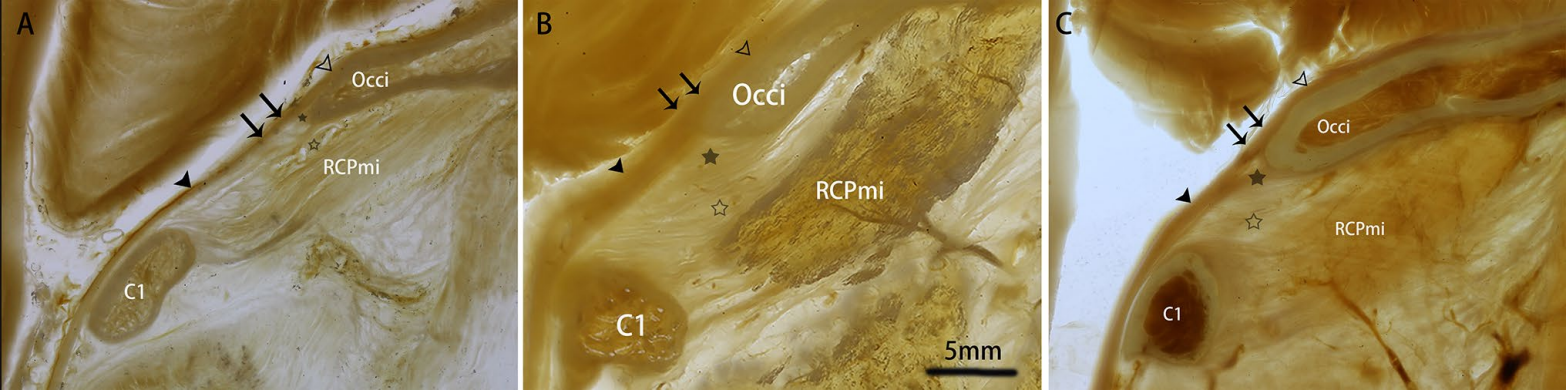
Images evidencing the fibers of different shapes and thicknesses emanating from the two distinct forms of the occipital bone, sharp vs. blunt. **A** the fibers from the occipital periosteum and the MDB were few and sparse; **B** fiber characteristics when the shape of the occipital bone is blunt; **C** fiber characteristics when the shape of the occipital bone is sharp. Occi, occipital bone, RCPmi, the rectus capitis posterior minor muscle; C1, atlas; hollow arrowhead, cerebral dura mater; solid arrowhead, spinal dura mater; double arrow, spinal dura mater; solid star, the fibers of the occipital periosteum; hollow star, the MDB

It was found that the cerebral dura mater, the periosteum of the occipital bone, and the MDB originating from the RCPmi muscle all contributed to the composition of the spinal dura mater at the CVJ. Except for one of the specimens, the fibers from the occipital periosteum and the MDB were few and sparsely distributed (Fig. 2A). Among these multiple components of the spinal dura mater, it was observed that the continuation of the cerebral dura mater was dense and thin, while the component from the occipital periosteum was dense and thick at the posterior atlanto-occipital interspace (Fig. 2). The outer layer of the SDM originated from the MDB and was observed to be looser than the other two components. Its fibers were observed to pass through the posterior atlanto-occipital interspace and to fuse with the SDM. Additionally, thicker fibers, originating from the occipital periosteum, were observed at the blunt brim type of the foramen magnum (Fig. 2B). The blunt brim type of the foramen magnum was observed in ten specimens or 48% of all specimens observed. Conversely, thinner fibers were observed at the sharper brim type of the foramen magnum (Fig. 2C). The sharper brim type of the foramen magnum was observed in 11 specimens or 52% of all specimens observed.

### Fusion characteristics of the three components of the SDM

**Fig. 3:**
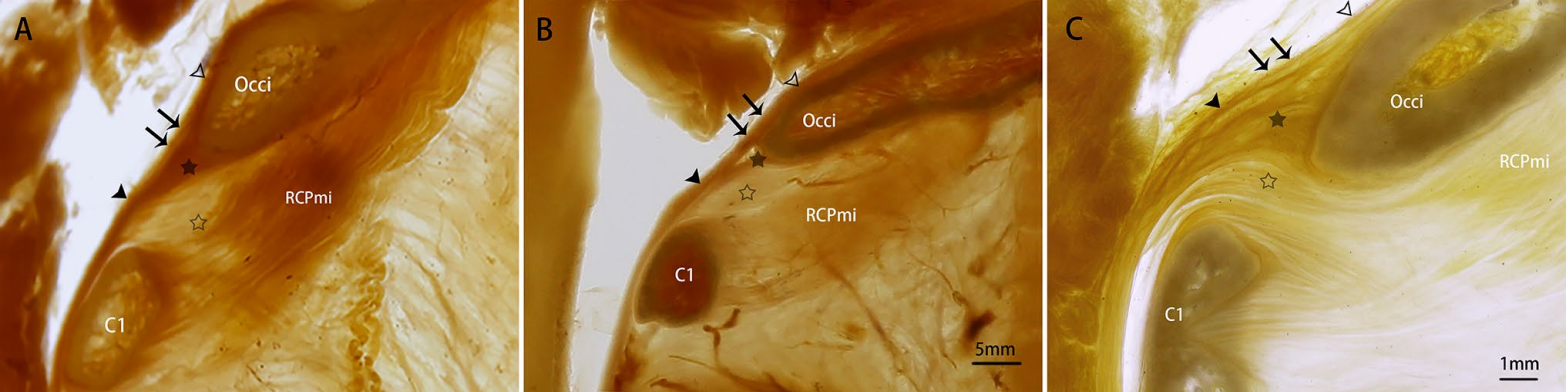
Images evidencing the confluence characteristics of the three types of constituent fibers. **A** no stratification between the fibers; **B** the distinct stratification between the fibers; **C**the multiple stratification between the fibers. Occi, occipital bone; RCPmi, the rectus capitis posterior minor muscle; C1, atlas; hollow arrowhead, cerebral dura mater; solid arrowhead, spinal dura mater; double arrow, spinal dura mater; solid star, the fibers of the occipital periosteum; hollow star, MDB; hollow arrow, the thickened spinal dura mater

In the sagittal sections of the SDM at the CVJ, we observed several variations of the confluence of multiple fibers originating from the cerebral dura mater, the occipital periosteum, and the MDB. Three distinct conditions were found: the fibers originating from the cerebral dura mater and from the occipital periosteum appeared to be tightly coalesced at the brim of the foramen magnum, with no obvious stratification observed between them (Fig. 3A). No obvious stratification was observed in seven specimens or 33% of all specimens observed. The fibers from the cerebral dura mater and the ones from the occipital periosteum are separated from each other beneath the brim of the foramen magnum. Thus, the loose interspace between these groups of fibers was only observed immediately below the brim of the foramen magnum (Fig. 3B), and the stratification between the fibers from the cerebral dura mater and the ones from the occipital periosteum was observed in 11 specimens or 52% of all specimens observed. These fibers originated from the cerebral dura mater, the occipital periosteum, and the MDB and were observed to be stratified at the posterior atlanto-occipital interspace. They were found to coalesce into one layer down to the level of the C1 (Fig. 3C), and the multiple stratification between the fibers originating from the cerebral dura mater, occipital periosteum, and MDB was observed in three specimens or 14% of all specimens observed.

## Discussion

In our study, the posterior wall of the spinal dura sac was observed in median and paramedian sagittal planes at the CVJ. We found that the SDM is composed of stratified layers of fibers, which are derived from three sources: the cerebral dura mater, occipital periosteum, and MDB. To our knowledge, this is the first time that the fibrous origin of the SDM has been precisely documented.

In previous studies [14, 28], scanning electron microscopy (SEM) and histological investigations observed that some of the MDB fibers originated from the ventral part of the RCPmi muscle. These fibers passed through the atlanto-occipital interspace and gradually merged into the SDM forming the superficial layer of the SDM. Some studies reported that the cerebral dura mater has two dural layers: the endosteal layer (outer layer) and the meningeal layer (inner layer) [3]. The extradural neural axis compartment (EDNAC) is an adipogenous zone located between the meningeal and endosteal layers of the dura. Parkinson developed the concept of EDNAC, which extends from the coccyx to the orbit. It runs along the neuraxis from the orbits down to the coccyx and contains fat, valveless veins, arteries, and nerves [23]. The inner and outer layers of EDNAC extend down to the suboccipital region and may form the periosteum and dura sources found in our study. The fibrous origin and composition of the cervical spinal dura mater were systematically observed and described in the present study. These previous researches support the point of our study from another aspect.

Our findings provide an explanation for Chauvet et al. suggesting that the midline sections of the spinal dura mater are thicker than the lateral sections [1]. Moreover, Feipel et al. [2] have suggested that cervical spine motion does not induce a significant strain on the cranial dura mater, to which this study provides anatomic clues. We found that at the CVJ, the innermost part of the SDM is the direct continuation of the cervical dura mater. Moreover, the majority of the middle fibers originated from the periosteum of the occipital bone, and the outer layer was observed to be composed of a small number of fibers originating from the MDB. Therefore, during cervical spine movement, the resulting stress placed on the spinal dura mater may be absorbed by the occipital periosteum and the suboccipital RCPMi muscle via the MDB.

In our study, the fibers originating from the cerebral dura mater, the occipital periosteum, and the MDB were observed to be composed of stratified layers of fibers at the posterior atlanto-occipital interspace. Chauvet D et al. [1] suggested that the distribution of blood vessels produced the stratification observed within the spinal dura mater. Based on our observations, we suggest that the different sources of fibers composing the spinal dura mater are the root cause of the stratification, and the blood vessels often appear between the fibers from the different sources. The findings of the present study should provide anatomical guidance or the surgical treatment of Chiari malformations (CM) and other cranio-cervical junction diseases. At present, the surgical treatment of CM primarily involves bone decompression, duraplasty, the resection of the tonsils of the cerebellum, and other methods [4, 11, 12, 22, 31]. These cervical surgical procedures often result in postoperative complications such as cerebrospinal fluid leakage, pseudomeningocele, hemorrhage, and reduced dural tension, due to occipital periosteum dissection, MDB removion, and improper dural dissection [9, 14, 18, 19, 24]. Therefore, we propose that during surgery, attention should be paid to the protection of the three sources of fibers of composing the spinal dura mater. When occipital fenestration is performed, the periosteum of at the margin of the foramen magnum should be protected to prevent dura damage and cerebrospinal fluid fistula formation. The results of the present study showed that the inner layer of the SDM is a direct continuation of the cervical dura mater, and the middle layer is mostly derived from the periosteum. Therefore, during surgical procedures, the periosteum located near the foramen magnum could be identified and dissected along the fibers to protect the inner layer of the SDM and thus prevent CSF fistula formation. When the cerebellar tonsillar tumor is resected [5], the periosteum of the occipital bone should be carefully stripped, and the dura mater should be carefully protected and re-sutured to the occipital bone to bear the changes in stress from the dura mater. Studies have shown that stress concentration caused by epidural adhesion is associated with spinal pain [15]. Intracranial nociception is mainly mediated by the trigeminal system [26]. We speculate that improper separation of the dura mater during surgery and destruction of the periosteum or MDB source of the dura mater may lead to local stress concentration on the dura mater during neck movement, stimulate the EDANC or intracranial blood vessels [27], and activate the trigeminal vascular system, resulting in headache and a series of neurogenic inflammations. Moreover, as the MDB has a dynamic effect on cerebrospinal fluid circulation [33], it should be protected during surgical operations.

## Conclusion

The spinal dura mater is a multi-layered structure composed of three different fiber types. These fiber types originated from the cerebral dura mater, periosteum located at the brim of the foramen magnum, and the myodural bridge that is associated with the rectus capitis posterior minor muscle.
